# Predicting stroke-associated pneumonia for acute ischemic stroke patients post intravenous thrombolysis: the post-IVT-SAP risk score

**DOI:** 10.3389/fmed.2026.1872375

**Published:** 2026-08-20

**Authors:** Daofeng Hu, Min Zhou, Lai Wei, Zhifeng Liu, Guoyong Zeng, Haoyi Ye, Jidong Peng, Yuping Zeng

**Affiliations:** 1 Department of Medical Imaging, Ganzhou People’s Hospital, The Affiliated Ganzhou Hospital, Jiangxi Medical College, Nanchang University, Ganzhou, China; 2 Department of Neurology, Ganzhou People’s Hospital, The Affiliated Ganzhou Hospital, Jiangxi Medical College, Ganzhou, China; 3 Department of Radiology, Shanghai Fourth People’s Hospital, School of Medicine, Tongji University, Shanghai, China; 4 Department of Medical Imaging, The Fourth Affiliated Hospital of Guangzhou Medical University, Guangzhou, China

**Keywords:** acute ischemic stroke (AIS), intravenous thrombolysis (IVT), post-IVT-SAP risk score (PISRS), predictive score, stroke-associated pneumonia (SAP)

## Abstract

**Introduction:**

Few prediction tools have been developed specifically for stroke-associated pneumonia (SAP) in patients with acute ischemic stroke (AIS) undergoing intravenous thrombolysis (IVT). This study aimed to develop and externally evaluate a simple risk score for SAP after IVT.

**Materials and methods:**

This multicenter retrospective study included 836 patients with AIS treated with IVT at three hospitals. Patients from two hospitals formed the training cohort, and those from the third hospital formed the external validation cohort. Univariable and multivariable logistic regression analyses were performed to identify independent predictors and develop a simple risk prediction score. Model discrimination was evaluated using receiver operating characteristic curve analysis.

**Results:**

Among 836 patients, 145 (17.3%) developed SAP. Age >75 years, atrial fibrillation, a higher pre-thrombolysis National Institutes of Health Stroke Scale score, and a lower pre-thrombolysis Alberta Stroke Program Early CT Score were independently associated with SAP and incorporated into the PISRS. The PISRS yielded an area under the curve of 0.718 in the training cohort and 0.801 (95% CI, 0.684–0.919) in the external validation cohort. Each 1-point increase in the PISRS was associated with increased odds of SAP within 7 days (OR = 2.623; 95% CI, 2.143–3.212).

**Conclusion:**

The PISRS is a simple admission-based score that may facilitate early risk stratification for SAP in patients with AIS undergoing IVT. Further prospective validation in larger and more diverse cohorts is required.

## 1 Introduction

Stroke-associated pneumonia (SAP) is a common complication of stroke and is associated with increased mortality, prolonged hospitalization, and unfavorable functional outcomes (1). It is generally defined as pneumonia occurring within 7 days after stroke onset in patients who are not receiving mechanical ventilation (2). The reported incidence of SAP ranges from approximately 14.0% to 27.8% (3, 4). However, early recognition remains challenging because clinical manifestations may be subtle during the initial stage after stroke. Early identification of patients at increased risk may enable closer respiratory surveillance and timely implementation of preventive measures, including aspiration precautions, swallowing assessment, and optimized oral care (5, 6).

Previous studies have identified multiple clinical and biological factors associated with SAP, including advanced age, male sex, vascular comorbidities, dysphagia, atrial fibrillation (AF), greater stroke severity assessed using the National Institutes of Health Stroke Scale (NIHSS), stroke-related immune dysfunction, and inflammatory responses (5–14). Several prediction models have consequently been developed to support early risk stratification. However, these models were derived from heterogeneous stroke populations, and their applicability to specific treatment settings remains uncertain. The performance and applicability of SAP prediction models may vary across different stroke populations and treatment settings, as clinical characteristics and potential risk factors may differ among these populations. For example, the ICH-LR2S2 score was developed specifically for patients with spontaneous intracerebral hemorrhage, highlighting the need to consider population characteristics when developing and validating SAP prediction models (15). Patients undergoing intravenous thrombolysis (IVT) represent a clinically distinct subgroup because they are selected according to specific eligibility criteria and receive reperfusion therapy within a defined therapeutic time window (16). Moreover, the risk factors associated with post-stroke infection may differ according to reperfusion strategies, as demonstrated in patients undergoing endovascular treatment (17). Although the A2DS2 score was developed and validated in large cohorts of patients with acute ischemic stroke and showed good predictive performance, it was not specifically derived from an IVT-treated population (18).

Therefore, this study aimed to identify factors independently associated with SAP in patients with acute ischemic stroke (AIS) undergoing IVT and to develop and externally evaluate a simple risk prediction model based on routinely available clinical, laboratory, and imaging variables. The model was intended to facilitate early risk stratification and complement routine clinical assessment rather than replace clinical judgment or serve as a stand-alone treatment rule.

## 2 Materials and methods

### 2.1 Ethics

This multicenter study was approved by the Ethics Committees of Ganzhou People’s Hospital, The Fourth Affiliated Hospital of Guangzhou Medical University, and the Tongji Hospital of Tongji University, and was exempted from obtaining informed consent from patients because it was a retrospective and observational study.

### 2.2 Study participants

**FIGURE 1:**
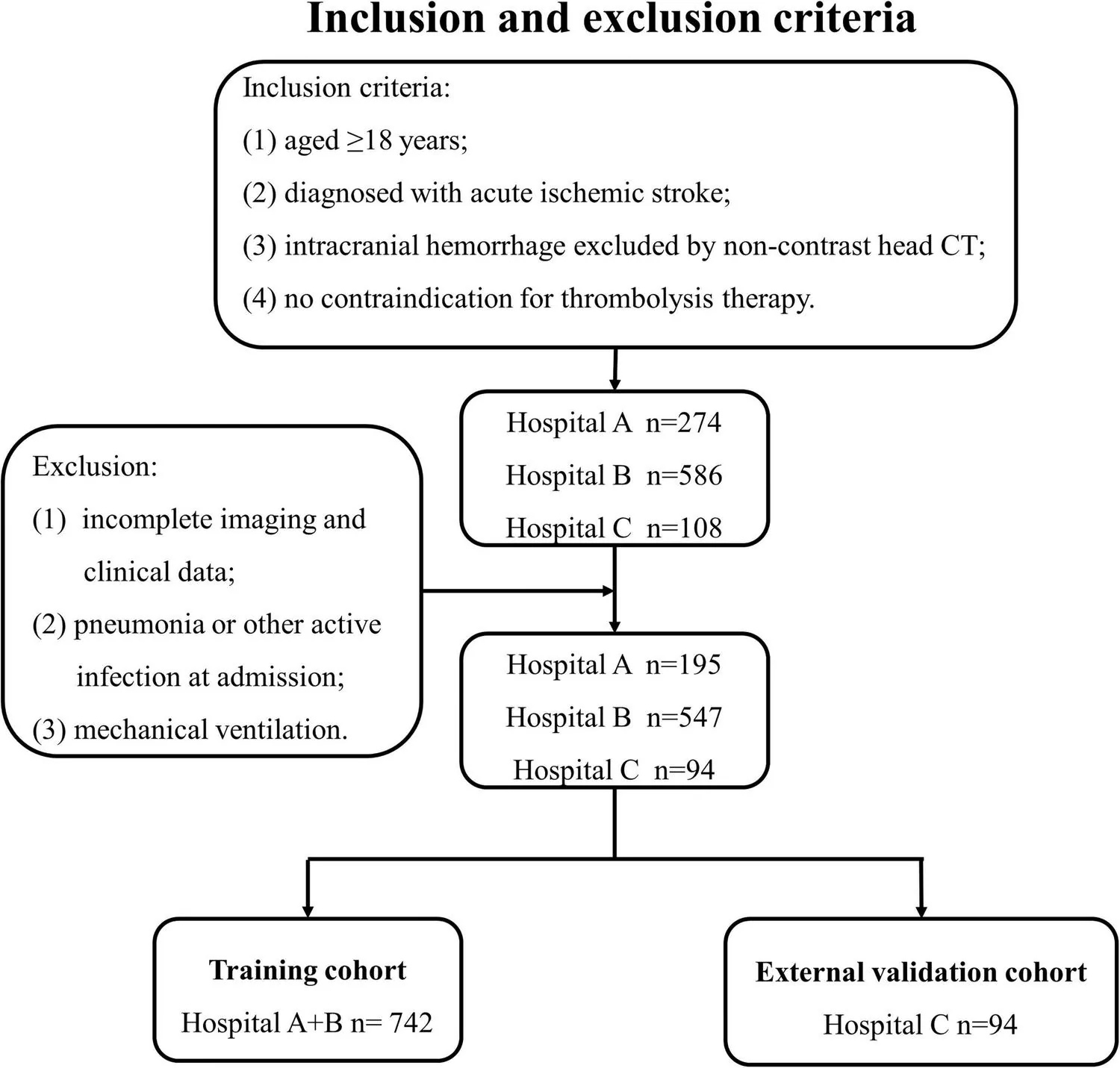
Inclusion and exclusion criteria.

Data were retrospectively collected on 836 patients with AIS who underwent IVT from January 1, 2020, to December 31, 2024, in three hospitals (Ganzhou People’s Hospital, Fourth Affiliated Hospital of Guangzhou Medical University, and Tongji Hospital of Tongji University). AIS patients who met the following criteria and accepted IVT were included in this study: (1) aged ≥18 years; (2) diagnosed with acute ischemic stroke; (3) intracranial hemorrhage excluded by non-contrast head CT; and (4) no contraindication for thrombolysis therapy. Patients with pre-existing pneumonia or active infection at admission, those receiving mechanical ventilation, and those with incomplete clinical information or unavailable chest CT images were excluded, as shown in Figure 1. Patient data from Ganzhou People’s Hospital and the Fourth Affiliated Hospital of Guangzhou Medical University were used as the training cohort to build the model, and patient data from Tongji Hospital of Tongji University were used as the external validation cohort to test the model performance.

### 2.3 Clinical data collection and imaging assessment

Clinical variables were retrospectively collected from medical records, including demographic characteristics, medical history, NIHSS scores, pre-thrombolysis CT images, complete blood counts, coagulation profiles, lipid levels, and other laboratory parameters. Age and NIHSS score were categorized according to classifications reported in previous studies. Age was classified as ≤75 and >75 years (18), whereas NIHSS scores were categorized into mild (0–9), moderate (10–19), and severe (≥20) neurological deficits (19–21). Pre-thrombolysis CT images were independently reviewed by three experienced neuroradiologists who were blinded to clinical outcomes and SAP status. The Alberta Stroke Program Early CT Score (ASPECTS) was used to evaluate early ischemic changes on baseline CT images. In cases of disagreement, a consensus was reached through discussion.

### 2.4 Diagnostic criteria for SAP

Post-IVT stroke-associated pneumonia (Post-IVT-SAP) was defined as a new lower respiratory tract infection occurring within 7 days after stroke onset in patients treated with intravenous thrombolysis. Patients with pre-existing pneumonia or other active infections at admission and those requiring mechanical ventilation were excluded. Post-IVT-SAP was diagnosed by experienced clinicians according to the modified Centers for Disease Control and Prevention criteria (2), based on an integrated assessment of clinical manifestations, laboratory findings, sputum cultures and chest imaging. The clinicians and radiologists responsible for SAP diagnosis were blinded to the candidate predictors and cohort allocation. Chest CT was routinely performed on days 1, 3, and 7 after thrombolysis, with additional examinations when pulmonary infection was clinically suspected. Sputum cultures were performed only when clinically indicated and were considered supportive rather than mandatory diagnostic evidence.

### 2.5 Statistical analysis

Statistical analyses were performed using IBM SPSS Statistics version 26.0 and R version 4.4.2. Patients with substantial missing clinical data or missing outcome data were excluded. Sporadic missing values in continuous variables were imputed using the series mean, whereas missing categorical predictor data were handled using available-case analysis. All statistical tests were two-sided, and *P* < 0.05 was considered statistically significant. Continuous variables were expressed as mean ± standard deviation or median with interquartile range, as appropriate, and compared using the independent-samples *t*-test or Mann–Whitney *U* test. Categorical variables were expressed as frequencies and percentages and compared using the Chi-square test or Fisher’s exact test, as appropriate.

Univariable logistic regression analysis was performed to identify factors associated with Post-IVT-SAP. Variables with *P* < 0.05 were entered into the multivariable logistic regression model, and adjusted odds ratios with 95% confidence intervals were reported. A simplified point-based score was empirically developed from the predictors retained in the final multivariable model. For continuous predictors retained in the final model, cut-off values for point assignment were determined using receiver operating characteristic curve analysis and the maximum Youden index. Dichotomous predictors were assigned 1 point when present, whereas predictors with ordered categories were assigned increasing points according to category severity. Model discrimination was evaluated using receiver operating characteristic curves (ROC) and areas under the curve (AUC) with 95% confidence intervals in the training and external validation cohorts. The optimal score threshold was determined using the maximum Youden index. Calibration was assessed using a calibration curve and the Hosmer–Lemeshow goodness-of-fit test. Decision curve analysis was performed in the training cohort to compare the net benefit of the simplified score with that of each independent predictor across a range of threshold probabilities.

## 3 Results

### 3.1 Demographic and clinical characteristics of the study population

A total of 836 patients were included in the study. The mean age was 65.6 ± 12.1 years, and 597 patients (71.4%) were male. Twenty-five variables were collected for analysis, including demographic characteristics, medical history, clinical variables, and laboratory findings. Among all patients, 145 (17.3%) developed SAP, whereas 691 (82.7%) did not. The incidence of SAP did not differ significantly between the training and external validation cohorts (*P* = 0.624). However, significant differences were observed between the two cohorts in 10 variables, including AF, pre-thrombolysis ASPECTS, onset-to-treatment time (OTT), and NLR (all *P* < 0.05), as shown in Table 1.

**TABLE 1:** Comparison of baseline data between patients in the training cohort and the external validation cohort.

Variable	Total (*n* = 836)	Training (*n* = 742)	External validation (*n* = 94)	*P* value
SAP	145 (17.3%)	127 (17.1%)	18 (19.1%)	0.624
Male, *n* (%)	597 (71.4%)	533 (71.8%)	64 (68.1%)	0.449
Age	65.57 ± 12.08	65.35 ± 12.04	67.29 ± 12.35	0.143
HTN, *n* (%)	542 (64.8%)	476 (64.2%)	66 (70.2%)	0.246
CHD, *n* (%)	79 (9.4%)	69 (9.3%)	10 (10.6%)	0.676
DM, *n* (%)	187 (22.4%)	153 (20.6%)	34 (36.2%)	0.001*
IS, *n* (%)	164 (19.6%)	147 (19.8%)	17 (18.1%)	0.691
AF, *n* (%)	75 (9.0%)	57 (7.7%)	18 (19.1%)	<0.001*
NIHSS	6 (3–11)	6 (3–11)	7 (3–12)	0.768
ASPECTS	9 (8–10)	9 (8–10)	10 (10–10)	<0.001*
OTT	150 (110.25–210)	150 (120–210)	116.5 (80–150)	<0.001*
DNT	50 (34–74)	50 (34–75)	50.5 (34.50–60)	0.266
NLR	3.11 (1.96–5.09)	3.19 (2.01–5.16)	2.61 (1.59–3.98)	<0.001*
NEUT%	68.65 (59.70–77.20)	69.20 (60.48–78.20)	65.9 (54.75–72.98)	<0.001*
LYMPH%	22.10 (15.30–30.48)	21.90 (15.00–29.90)	25.25 (18.33–34.25)	0.002*
MPV	9.70 (9.10–10.40)	9.70 (9.10–10.30)	9.65 (8.90–10.63)	0.702
PDW	12.10 (10.13–16.00)	11.50 (10.00–15.70)	16.20 (15.88–16.50)	<0.001*
PT	11.10 (10.50–11.70)	11.10 (10.60–11.70)	11.10 (10.30–11.83)	0.415
INR	0.95 (0.91–1.01)	0.95 (0.91–1.01)	0.97 (0.89–1.03)	0.725
APTT	25.40 (23.50–27.60)	25.00 (23.30–27.30)	27.80 (26.48–29.23)	0.060
TT	17.10 (16.20–18.20)	16.95 (16.00–18.10)	17.90 (17.28–18.90)	<0.001*
GLU	6.20 (5.03–8.01)	6.11 (4.99–7.79)	7.03 (5.50–9.28)	0.001*
HbA1c	6.10 (5.70–6.70)	6.00 (5.70–6.62)	6.60 (6.07–7.90)	0.075
FIB	2.90 (2.43–3.45)	2.93 (2.46–3.45)	2.64 (2.33–3.31)	0.067
SBP	153 (140–169)	152 (140–169)	154.5 (139.75–168.25)	0.686
DBP	86 (79–96)	86 (79–96)	89.5 (78–101)	0.198

Data are presented as mean ± standard deviation, median (interquartile range), or *n* (%), as appropriate. *P* < 0.05 was considered statistically significant. All laboratory samples were collected at admission. HTN, hypertension; CHD, coronary heart disease; DM, diabetes mellitus; IS, ischemic stroke; AF, atrial fibrillation; NIHSS, National Institutes of Health Stroke Scale (before thrombolysis); ASPECTS, the Alberta Stroke Program Early CT Score (before thrombolysis); OTT, Onset to Treatment Time; DNT, Door to Needle Time; NLR, neutrophil-to-lymphocyte ratio; NEUT%, neutrophil percentage; LYMPH%, lymphocyte percentage; MPV, mean platelet volume (fL); PDW, platelet distribution width (fL); PT, prothrombin time (sec); INR, international normalized ratio; APTT, activated partial thromboplastin time (sec); TT, thrombin time (sec); GLU, glucose (mmol/L); HbA1c, glycated hemoglobin; FIB, fibrinogen (g/L); SBP, systolic blood pressure; DBP, diastolic blood pressure.

### 3.2 Univariable analysis

In the training cohort, univariable logistic regression analysis identified several factors associated with Post-IVT-SAP (Table 2). Compared with patients aged ≤75 years, those aged >75 years had higher odds of developing Post-IVT-SAP (OR = 1.876, 95% CI, 1.276–2.759). CHD (OR = 1.988, 95% CI, 1.128–3.504) and AF (OR = 3.779, 95% CI, 2.140–6.675) were also associated with increased odds of Post-IVT-SAP. Using an NIHSS score of 0–9 as the reference, the corresponding ORs were 3.335 (95% CI, 2.151–5.232) for scores of 10–19 and 9.045 (95% CI, 4.964–16.465) for scores ≥20. Higher NLR (OR = 1.036, 95% CI, 1.011–1.063), NEUT% (OR = 1.034, 95% CI, 1.017–1.051), and FIB levels (OR = 1.238, 95% CI, 1.013–1.514) were associated with increased odds of Post-IVT-SAP. By contrast, higher pre-thrombolysis ASPECTS (OR = 0.763, 95% CI, 0.672–0.866) and LYMPH% (OR = 0.955, 95% CI, 0.936–0.974) were associated with lower odds of Post-IVT-SAP. No significant associations were observed for HTN, DM, or MPV.

**TABLE 2:** Training cohort: univariable analysis of SAP variables predicting the occurrence of SAP within 7 days.

Variable	OR	95% CI	*P* value
Male	0.722	0.480–1.086	0.118
Age >75 years	1.876	1.276–2.759	0.001*
HTN	0.693	0.457–1.051	0.084
CHD	1.988	1.128–3.504	0.018*
DM	1.448	0.929–2.257	0.102
IS	1.465	0.935–2.294	0.096
AF	3.779	2.140–6.675	<0.001*
NIHSS			
0–9	Reference		
10–19	3.335	2.151–5.232	<0.001*
≥20	9.045	4.964–16.465	<0.001*
ASPECTS	0.763	0.672–0.866	<0.001*
OTT	1.000	0.999–1.001	0.752
DNT	1.001	0.998–1.003	0.510
NLR	1.036	1.011–1.063	0.005*
NEUT%	1.034	1.017–1.051	<0.001*
LYMPH%	0.955	0.936–0.974	<0.001*
MPV	1.006	0.969–1.044	0.753
PDW	1.011	0.916–1.116	0.828
PT	1.062	0.906–1.246	0.457
INR	2.214	0.329–14.874	0.414
APTT	0.971	0.928–1.017	0.220
TT	0.989	0.915–1.070	0.788
GLU	1.048	0.988–1.111	0.118
HbA1c	0.956	0.836–1.093	0.510
FIB	1.238	1.013–1.514	0.037*
SBP	1.006	0.998–1.014	0.159
DBP	1.002	0.988–1.016	0.758

All laboratory test samples were collected from the time of admission. HTN, hypertension; CHD, coronary heart disease; DM, diabetes mellitus; IS, ischemic stroke; AF, atrial fibrillation; NIHSS, National Institutes of Health Stroke Scale (before thrombolysis); ASPECTS, the Alberta Stroke Program Early CT Score (before thrombolysis); NLR, neutrophil-to-lymphocyte ratio; NEUT%, neutrophil percentage; LYMPH%, lymphocyte percentage; MPV, mean platelet volume (fL); PDW, platelet distribution width (fL); PT, prothrombin time (sec); INR, international normalized ratio; APTT, activated partial thromboplastin time (sec); TT, thrombin time (sec); GLU, glucose (mmol/L); HbA1c, glycated hemoglobin; FIB, fibrinogen (g/L); SBP, systolic blood pressure; DBP, diastolic blood pressure.

### 3.3 Multivariable analysis

**FIGURE 2:**
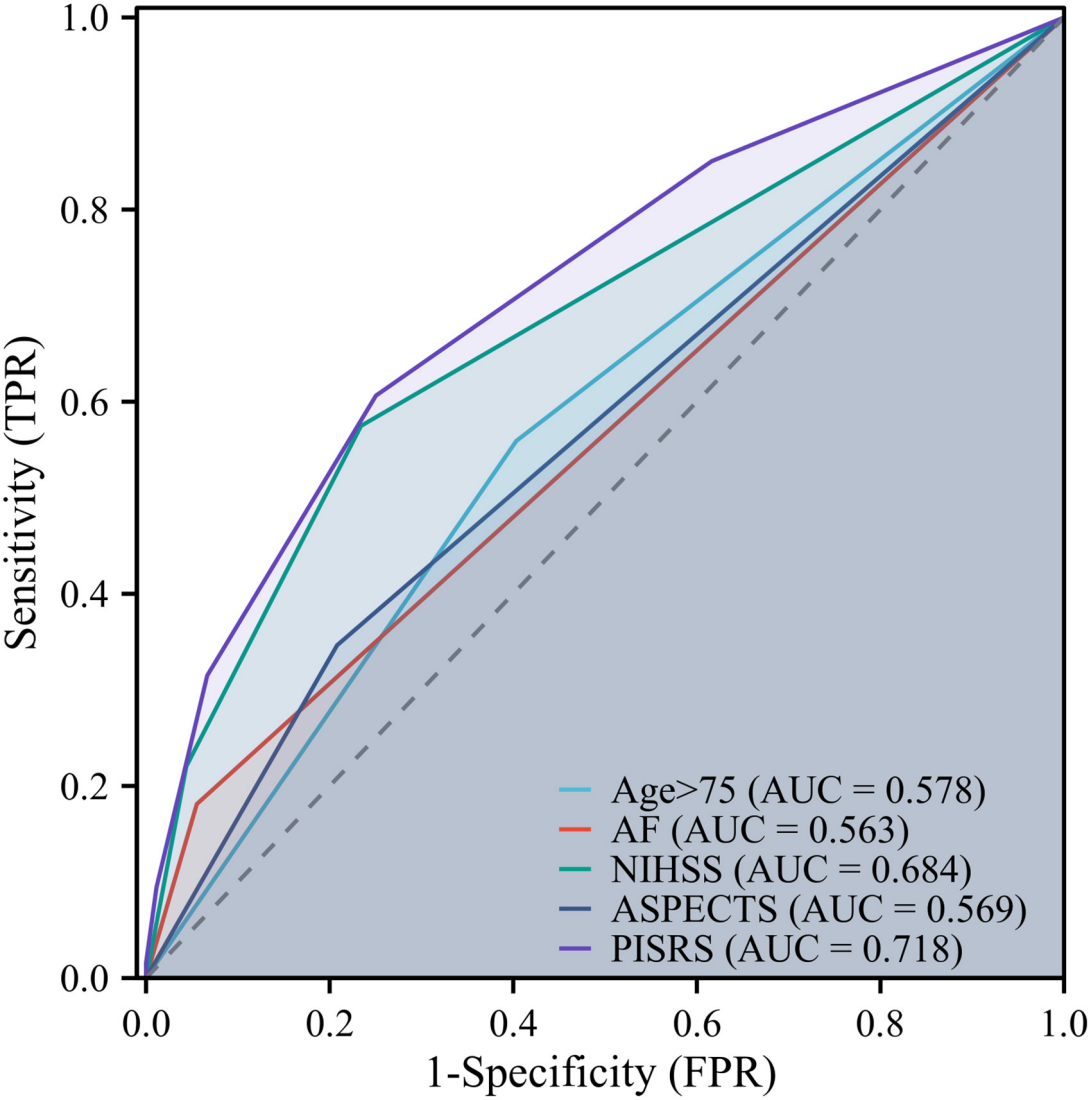
Receiver operating characteristic curves of the individual predictors and the PISRS for predicting Post-IVT-SAP in the training cohort.

In the training cohort, multivariable logistic regression analysis showed that age >75 years (adjusted OR = 1.640, 95% CI, 1.066–2.523), AF (adjusted OR = 2.689, 95% CI, 1.414–5.113), and higher pre-thrombolysis NIHSS scores remained independently associated with increased odds of Post-IVT-SAP. Using an NIHSS score of 0–9 as the reference, the adjusted ORs were 2.529 (95% CI, 1.575–4.061) for scores of 10–19 and 5.733 (95% CI, 3.023–10.876) for scores ≥20. By contrast, a higher pre-thrombolysis ASPECTS was independently associated with lower odds of Post-IVT-SAP (adjusted OR = 0.791, 95% CI, 0.688–0.909), as shown in Table 3. Among the four predictors retained in the final model, pre-thrombolysis NIHSS showed the greatest discriminative ability, with an AUC of 0.684 (Figure 2).

**TABLE 3:** Training cohort: multivariable analysis of SAP variables predicting the occurrence of SAP within 7 days.

Variable	β	OR	95% CI	*p value*
Age >75 years	0.495	1.640	1.066–2.523	0.024*
CHD	0.426	1.532	0.808–2.904	0.192
AF	0.989	2.689	1.414–5.113	0.003*
NIHSS				
0–9	Reference			
10–19	0.928	2.529	1.575–4.061	<0.001*
≥20	1.746	5.733	3.023–10.876	<0.001*
ASPECTS	−0.234	0.791	0.688–0.909	0.001*
NLR	0.016	1.016	0.982–1.051	0.361
NEUT%	−0.009	0.991	0.954–1.030	0.649
LYMPH%	−0.039	0.962	0.916–1.011	0.125
FIB	0.127	1.135	0.907–1.421	0.269

All laboratory test samples were collected from the time of admission. CHD, coronary heart disease; AF, atrial fibrillation; NIHSS, National Institutes of Health Stroke Scale (before thrombolysis); ASPECTS, the Alberta Stroke Program Early CT Score (before thrombolysis); NLR, neutrophil-to-lymphocyte ratio; NEUT%, neutrophil percentage; LYMPH%, lymphocyte percentage; FIB, fibrinogen.

### 3.4 Establishment of the Post-IVT-SAP risk score (PISRS)

**FIGURE 3:**
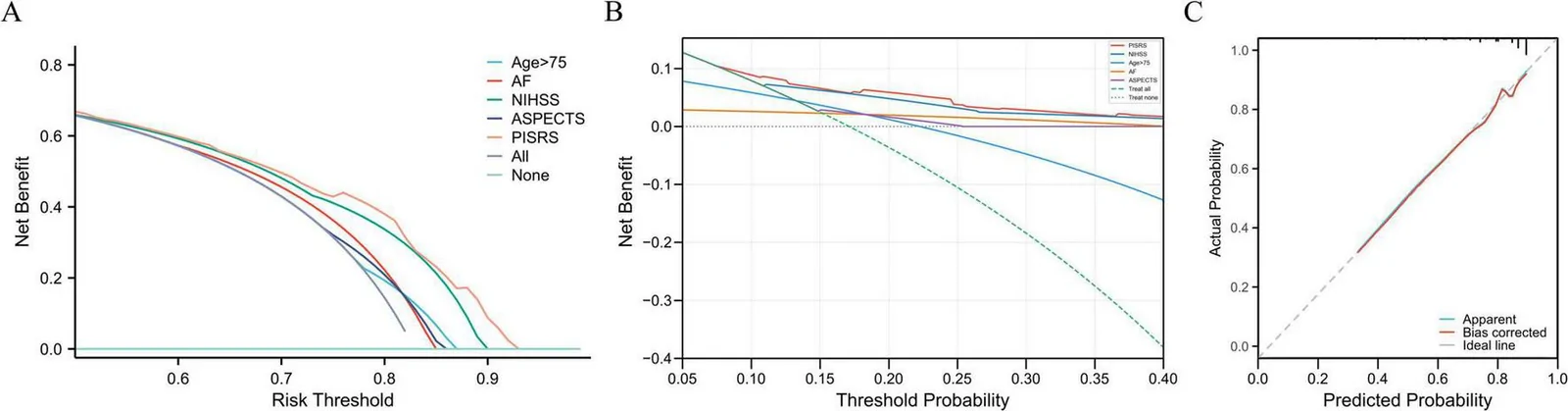
Decision curve and calibration analyses of the PISRS in the training cohort. **(A)** Decision curve analysis comparing the PISRS with individual predictors at higher risk thresholds. **(B)** Decision curve analysis within the 5%–40% risk-threshold range; the green dashed and gray dotted lines represent the treat-all and treat-none strategies, respectively. **(C)** Apparent and bias-corrected calibration curves of the PISRS.

Based on the results of the multivariable logistic regression analysis, the PISRS was developed as a simplified point-based score using four independent predictors: age, AF, NIHSS, and ASPECTS (Table 4). ROC analysis of the PISRS in the training cohort yielded an AUC of 0.718 (Figure 2). The diagnostic performance of the PISRS at different cutoff values, including sensitivity, specificity, PPV, NPV, and likelihood ratios, is summarized in Supplementary Table 1. Decision curve analysis demonstrated the potential clinical utility of the PISRS for SAP risk stratification, with greater net benefit observed compared with individual predictors and the treat-all and treat-none strategies (Figures 3A, B). The calibration curve demonstrated satisfactory agreement between the predicted and observed probabilities of Post-IVT-SAP in the training cohort (Figure 3C). The Hosmer–Lemeshow goodness-of-fit test showed no significant difference between the observed and predicted outcomes (*P* = 0.9756).

**TABLE 4:** The post-IVT-SAP risk score.

Variables	Points (0–5)
Age	
≤75	0
>75	1
History of AF	
No	0
Yes	1
Before thrombolysis NIHSS	
0–9	0
10–19	1
≥20	2
Before thrombolysis ASPECTS	
≥8	0
<8	1

AF, atrial fibrillation; NIHSS, National Institutes of Health Stroke Scale; ASPECTS, the Alberta Stroke Program Early CT Score.

### 3.5 External validation of PISRS and relationship between PISRS and occurrence of SAP

**FIGURE 4:**
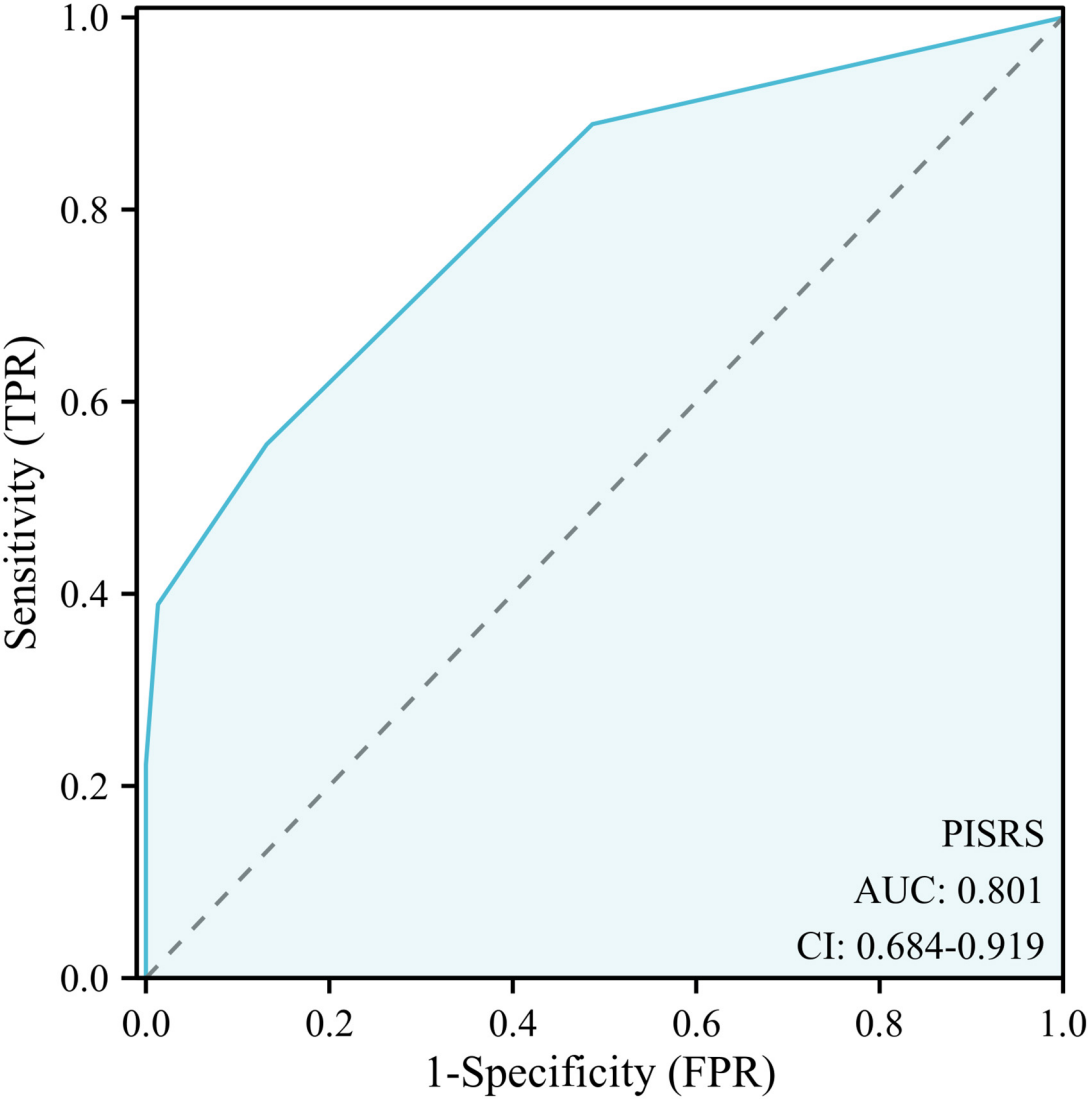
Receiver operating characteristic curve of the PISRS for predicting Post-IVT-SAP in the external validation cohort.

**FIGURE 5:**
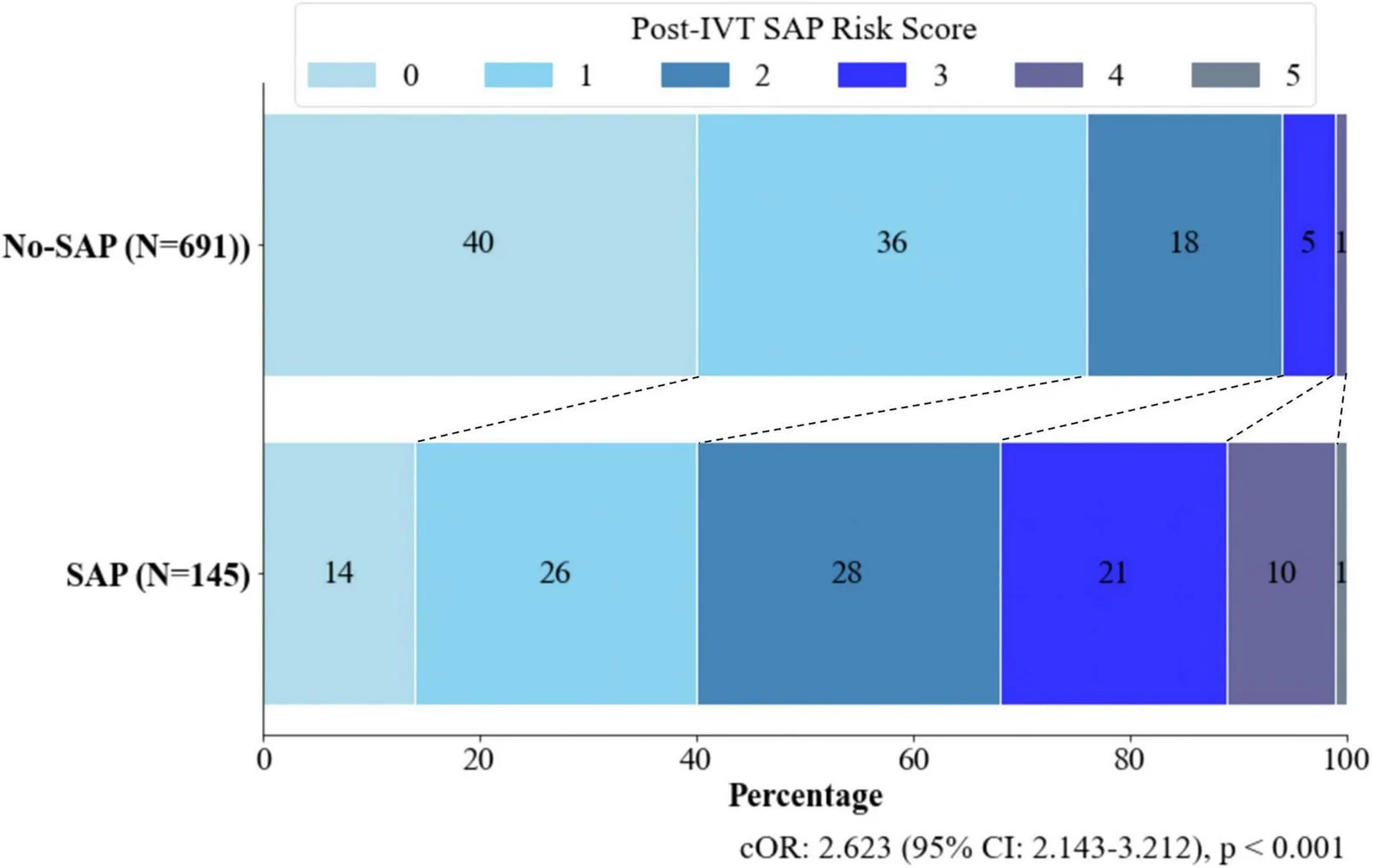
Association between the PISRS and the occurrence of SAP in patients with AIS undergoing IVT.

In the external validation cohort, ROC analysis of the PISRS yielded an AUC of 0.801 (95% CI, 0.684–0.919) (Figure 4). The logistic regression model showed slightly higher AUC values than the PISRS; however, the difference was not statistically significant in the external validation cohort (AUC difference: 0.024, *P* = 0.155; Supplementary Table 3). Using a cutoff value of 2 points, 60.0% of patients with SAP had a PISRS of ≥2, compared with 24.0% of those without SAP (*P* < 0.05). In the overall cohort, each 1-point increase in the PISRS was associated with a 2.623-fold increase in the odds of developing SAP within 7 days (OR = 2.623; 95% CI, 2.143–3.212; Figure 5).

## 4 Discussion

In this multicenter retrospective study of patients with acute ischemic stroke treated with intravenous thrombolysis, older age, AF, a higher pre-thrombolysis NIHSS score, and a lower pre-thrombolysis ASPECTS were independently associated with the development of Post-IVT-SAP. These four predictors were incorporated into the PISRS, a simple 0–5-point risk score based entirely on variables available at admission. Each 1-point increase in the PISRS was associated with a 2.623-fold increase in the odds of developing SAP within 7 days. The PISRS yielded an AUC of 0.718 in the training cohort and 0.801 in the external validation cohort, suggesting that it may provide a practical approach to early risk stratification after IVT.

The PISRS uses explicitly defined scoring items and can be calculated rapidly without complex computation or additional laboratory testing. This structure may reduce variability arising from inter-observer variability and facilitate implementation in routine practice. By contrast, machine-learning models can integrate multiple heterogeneous variables and capture complex nonlinear relationships, potentially achieving higher predictive performance. For example, Abujaber et al. incorporated stroke severity, admission location, and Glasgow Coma Scale (GCS) score into an artificial neural network for SAP prediction and reported an AUC of 0.94 (22). However, the implementation of such models in routine clinical practice may be limited by computational requirements, reduced interpretability, and concerns regarding their “black-box” nature. Conventional clinical scales, such as the NIHSS and GCS, remain widely used because their component items have clear clinical meanings and their contribution to the final score can be readily understood. Accordingly, the principal advantage of the PISRS lies in its simplicity, transparency, and immediate availability rather than in superior predictive accuracy.

Advanced age is a well-recognized risk factor for SAP. Age-related atrophy and weakening of the oral and pharyngeal musculature, together with impaired swallowing coordination, may increase susceptibility to aspiration (23, 24). Higher NIHSS scores and lower ASPECTS generally reflect greater neurological impairment and a larger burden of ischemic injury. In addition, injury to the swallowing center or corticobulbar pathways may lead to delayed swallowing, reduced laryngeal elevation, and cricopharyngeal incoordination. Severe hemiparesis, prolonged bed rest, and reduced mobility may further promote pulmonary secretion retention. These mechanisms may explain the independent associations of higher NIHSS scores and lower ASPECTS with Post-IVT-SAP. Atrial thrombi may occlude proximal cerebral arteries and produce extensive or multifocal cerebral infarction, resulting in more severe neurological deficits, impaired mobility, and prolonged hospitalization (25). When the embolic source is not adequately controlled, early recurrent cerebral embolism may further increase the burden of neurological injury. These factors may contribute to the increased risk of SAP among patients with AF.

The association between AIS and infection may also be partly mediated by stroke-induced immunodepression syndrome. Activation of sympathetic, parasympathetic, and hypothalamic–pituitary–adrenal pathways after AIS may induce systemic immunosuppression (11, 26). These responses may contribute to rapid alterations in circulating lymphocyte populations, including reductions in selected T-cell subsets and impaired cell-mediated immunity (27). Post-stroke immune dysregulation may also impair pulmonary innate immune defense, thereby increasing susceptibility to respiratory infection (28). In the univariable analysis, CHD, NLR, NEUT%, LYMPH%, and FIB were significantly associated with Post-IVT-SAP; however, these associations were attenuated and were no longer statistically significant after multivariable adjustment. Cerebral and coronary atherosclerosis share several vascular risk factors, which may partly explain the univariable association between CHD and SAP. The laboratory variables reflect, to varying degrees, systemic inflammation and post-stroke immunodepression. Increased NEUT% and FIB levels may indicate an enhanced inflammatory response, whereas reduced LYMPH% is consistent with stroke-induced immunosuppression. Li et al. reported that higher FIB levels were associated with an increased risk of SAP (OR = 1.53, 95% CI, 1.18–1.99) (29).

NLR is derived from neutrophil and lymphocyte measurements, while NEUT% and LYMPH% are compositionally related. Their simultaneous inclusion in the multivariable model may therefore introduce information overlap and potential collinearity, resulting in inflated standard errors and attenuated individual coefficients. FIB may also overlap with information related to systemic inflammation and stroke severity. Furthermore, because blood samples were obtained early after stroke onset and before thrombolysis, systemic inflammatory and immunosuppressive responses may not have been fully established at the time of measurement. Therefore, the loss of statistical significance does not exclude the potential biological relevance of these variables but indicates that they did not provide independent predictive information beyond the variables retained in the final model. Inflammatory biomarkers have nevertheless shown predictive value for SAP across different stroke populations. In a prospective multicenter study of patients with intracerebral hemorrhage, Wang et al. compared several inflammatory indices and found that NLR had the highest predictive value for SAP, with an AUC of 0.748 (95% CI, 0.695–0.801) (30). Although NLR was not retained in the PISRS, it remained significantly associated with SAP in the univariable analysis. However, laboratory-based prediction models may be affected by differences in analytical instruments, reagents, sampling procedures, and testing protocols across institutions. Their transportability therefore requires further evaluation in larger multicenter cohorts. In comparison, the PISRS is based on routinely available clinical and imaging variables that are less susceptible to interlaboratory variation, which may facilitate broader application.

The external validation cohort showed a higher AUC than the training cohort. This difference may partly reflect sampling variability due to the relatively small sample size of the external validation cohort (*n* = 94, including 18 SAP events), as reflected by the wide 95% CI of the AUC (0.684–0.919), which overlapped with the AUC estimate of the training cohort (AUC = 0.718). Therefore, the observed difference in discrimination should be interpreted cautiously. Calibration assessment showed comparable overall prediction error between the training and external validation cohorts, with Brier scores of 0.1234 and 0.1159, respectively. However, the calibration intercept and slope in the external validation cohort deviated from the ideal values, suggesting potential calibration instability (Supplementary Table 4), which may be related to the limited sample size and differences in case mix between cohorts. Model discrimination and calibration are influenced by case mix. The external validation cohort differed from the training cohort in several characteristics, including the prevalence of AF, ASPECTS, onset-to-treatment time, and inflammatory variables, which may have affected the distribution of predictors and the separation between patients with and without SAP. Furthermore, differences in patient selection and measurement procedures between centers may also have contributed to variation in model performance. Further evaluation in larger multicenter cohorts would help to confirm the generalizability and stability of the model.

Early chest CT may provide more direct evidence of evolving pulmonary infection or aspiration-related pulmonary abnormalities. De Jonge et al. reported that signs of infection on admission chest CT were associated with an increased risk of subsequent SAP (OR = 5.3, 95% CI, 1.9–14.5) (31). However, subtle or limited ground-glass opacities may be interpreted differently among physicians, and image quality may be affected by partial-volume effects, respiratory motion, and inadequate breath-holding. Moreover, chest CT is not routinely required before IVT and could delay reperfusion treatment. Therefore, although early chest imaging may contribute useful information, it may not be suitable as a universally available predictor for immediate pre-thrombolysis risk assessment.

Previous SAP prediction models have demonstrated favorable performance; however, most were developed in heterogeneous stroke populations and differ in predictor requirements and clinical applicability. The A2DS2 score incorporates age, AF, dysphagia, male sex, and NIHSS, achieving a C-statistic of 0.84 with subsequent external validation (18). However, it was developed in a general AIS population rather than an IVT-specific cohort, and dysphagia assessment may introduce additional clinical evaluation requirements during the acute phase. The AIS-APS incorporates 11 clinical variables and achieved AUCs of 0.785 and 0.792 in internal and external validation cohorts, respectively (32), but its extensive data requirements may limit rapid application in acute stroke workflows. PANTHERIS achieved an AUC of 0.85 with internal validation but lacked external validation in the original study (33). Although the IVT-specific A2DS2-MFP model showed favorable performance, its reliance on dysphagia assessment, laboratory tests, 24-h imaging, and equation-based calculation may limit rapid clinical application (34). Machine learning approaches, such as the XGBoost model, have also been explored for SAP prediction and achieved an AUC of 0.841 using six clinical variables (35). However, these models generally require complex computational procedures, which may limit their direct implementation in routine clinical workflows. In contrast, the PISRS was specifically developed for AIS patients undergoing IVT and requires only four readily available admission variables (age, AF, pre-thrombolysis NIHSS, and baseline ASPECTS). The score can be easily calculated without additional laboratory testing, delayed imaging, or complex computation, and was further evaluated in an independent external cohort. Therefore, the potential clinical value of the PISRS lies in its simplicity, transparency, and applicability for early SAP risk stratification rather than superior discrimination over all existing models.

Decision curve analysis suggested that the PISRS provided greater net benefit than each individual predictor and the treat-all and treat-none strategies across most threshold probabilities above 60%. However, this relatively high threshold range may represent a selective clinical decision context, and the clinical utility of the PISRS should be prospectively evaluated. A total score of ≥2 may be used to identify patients who require closer respiratory surveillance, swallowing and aspiration-risk assessment, protocolized oral care, appropriate positioning and nutritional management, and early mobilization when clinically appropriate. Nevertheless, the PISRS should be regarded as an admission screening and risk-stratification aid rather than as a stand-alone treatment rule. Because this cutoff was not prospectively linked to a specific intervention pathway or improved clinical outcomes, management decisions should continue to be based on the overall clinical assessment and local protocols. In particular, the PISRS alone should not be used to initiate prophylactic antibiotic therapy.

This study has several limitations. First, although 836 patients from three centers were included, the retrospective design may have introduced selection bias, information bias, and unmeasured confounding. Second, internal validation was not performed because the development cohort was not further split into training and internal validation subsets. Given the limited sample size and number of SAP events, all available data from the two development centers were used for model construction to improve parameter estimation stability. Although an independent external validation cohort was included, its relatively small size and limited number of SAP events may have affected the precision of performance estimates. Further validation in larger and more diverse multicenter cohorts is warranted to confirm the robustness and generalizability of the PISRS. Finally, long-term functional outcomes were not systematically assessed, precluding evaluation of the impact of SAP on subsequent functional prognosis.

## 5 Conclusion

We developed and externally evaluated the PISRS, a simple admission-based scoring system for predicting SAP within 7 days in patients with AIS undergoing IVT. The PISRS may facilitate rapid risk stratification and early identification of patients at increased risk of SAP. Further prospective validation in larger and more diverse cohorts is required before its routine clinical implementation.

## Data Availability

The data that supports the findings of this study are available on request from the corresponding author.
