# Supplementary material for: Predicting stroke-associated pneumonia for acute ischemic stroke patients post intravenous thrombolysis: the post-IVT-SAP risk score
Source: Front Med (Lausanne). 2026 Aug 20;13:1872375. doi: 10.3389/fmed.2026.1872375 (PMC13542813; doi:10.3389/fmed.2026.1872375)
Supplement: Supplementary file 1 [file Table_1.docx]

**Supplementary Table S1. Diagnostic performance of PISRS at different cutoff values in the Training cohort**

| **PISRS cutoff** | **Sensitivity, % (95% CI)** | **Specificity, % (95% CI)** | **PPV, % (95% CI)** | **NPV, % (95% CI)** | **LR+ (95% CI)** | **LR- (95% CI)** |
| --- | --- | --- | --- | --- | --- | --- |
| >=1 | 85.0 (77.8-90.2) | 38.4 (34.6-42.3) | 22.2 (18.7-26.1) | 92.5 (88.7-95.2) | 1.38 (1.25-1.52) | 0.39 (0.25-0.60) |
| **>=2** | **60.6 (51.9-68.7)** | **75.0 (71.4-78.2)** | **33.3 (27.6-39.6)** | **90.2 (87.3-92.5)** | **2.42 (1.99-2.94)** | **0.53 (0.42-0.65)** |
| >=3 | 31.5 (24.1-40.0) | 93.3 (91.1-95.0) | 49.4 (38.8-60.0) | 86.8 (84.0-89.2) | 4.72 (3.19-6.99) | 0.73 (0.65-0.83) |
| >=4 | 9.4 (5.5-15.8) | 98.9 (97.7-99.4) | 63.2 (41.0-80.9) | 84.1 (81.2-86.6) | 8.30 (3.33-20.67) | 0.92 (0.87-0.97) |
| >=5 | 1.6 (0.4-5.6) | 100.0 (99.4-100.0) | 100.0 (34.2-100.0) | 83.1 (80.2-85.6) | NA | 0.98 (NA) |

CI = confidence interval; LR+ = positive likelihood ratio; LR- = negative likelihood ratio; NPV = negative predictive value; PPV = positive predictive value.

**Supplementary Table S2. Diagnostic performance of PISRS at different cutoff values in the External validation cohort**

| **PISRS cutoff** | **Sensitivity, % (95% CI)** | **Specificity, % (95% CI)** | **PPV, % (95% CI)** | **NPV, % (95% CI)** | **LR+ (95% CI)** | **LR- (95% CI)** |
| --- | --- | --- | --- | --- | --- | --- |
| >=1 | 88.9 (67.2-96.9) | 51.3 (40.3-62.2) | 30.2 (19.5-43.5) | 95.1 (83.9-98.7) | 1.83 (1.38-2.42) | 0.22 (0.06-0.81) |
| **>=2** | **55.6 (33.7-75.4)** | **86.8 (77.4-92.7)** | **50.0 (29.9-70.1)** | **89.2 (80.1-94.4)** | **4.22 (2.08-8.59)** | **0.51 (0.30-0.86)** |
| >=3 | 38.9 (20.3-61.4) | 98.7 (92.9-99.8) | 87.5 (52.9-97.8) | 87.2 (78.5-92.7) | 29.56 (3.88-225.34) | 0.62 (0.43-0.90) |
| >=4 | 22.2 (9.0-45.2) | 100.0 (95.2-100.0) | 100.0 (51.0-100.0) | 84.4 (75.6-90.5) | NA | 0.78 (NA) |
| >=5 | NA | 100.0 (95.2-100.0) | NA | 80.9 (71.7-87.5) | NA | 1.00 (NA) |

CI = confidence interval; LR+ = positive likelihood ratio; LR- = negative likelihood ratio; NPV = negative predictive value; PPV = positive predictive value.

**Supplementary Table S3. Comparison of discriminative performance between the Logistic model and PISRS**

| **Cohort** | **Patients, n** | **SAP events, n** | **Logistic model AUC (95% CI)** | **PISRS AUC (95% CI)** | **AUC difference (95% CI)** | **P value** |
| --- | --- | --- | --- | --- | --- | --- |
| Training | 742 | 127 | 0.729 (0.679-0.780) | 0.718 (0.668-0.768) | 0.011 (0.001-0.021) | 0.031 |
| External validation | 94 | 18 | 0.825 (0.709-0.941) | 0.801 (0.684-0.919) | 0.024 (-0.009-0.057) | 0.155 |

AUCs and 95% CIs were estimated using the DeLong method, and paired AUCs were compared using the DeLong test. AUC = area under the receiver operating characteristic curve; CI = confidence interval; SAP = stroke-associated pneumonia.

**Supplementary Table S4. Calibration performance of the PISRS model in the training and validation cohorts.**

| **Model** | **Cohort** | **Brier score** | **Calibration intercept** | **Calibration slope** |
| --- | --- | --- | --- | --- |
| **PISRS** | **Training cohort** | **0.1234** | **-0.0176** | **0.9873** |
|  | **External validation** | **0.1159** | **1.3103** | **1.6159** |
